# Capacitance-Voltage Characteristics of Thin-film Transistors Fabricated with Solution-Processed Semiconducting Carbon Nanotube Networks

**DOI:** 10.1186/s11671-015-0999-8

**Published:** 2015-07-15

**Authors:** Le Cai, Suoming Zhang, Jinshui Miao, Qinqin Wei, Chuan Wang

**Affiliations:** Department of Electrical and Computing Engineering, Michigan State University, East Lansing, MI 48824 USA

**Keywords:** Carbon nanotube network, Thin-film transistor, Gate capacitance, Interface trap density

## Abstract

We report the capacitance-voltage (C-V) measurements on thin-film transistors (TFTs) using solution-processed semiconducting carbon nanotube networks with different densities and channel lengths. From the measured C-V characteristics, gate capacitance and field-effect mobility (up to ~50 cm^2^ V^−1^ s^−1^) of the TFTs were evaluated with better precision compared with the results obtained from calculated gate capacitance. The C-V characteristics measured under different frequencies further enabled the extraction and analysis of the interface trap density at the nanotube-dielectric layer interface, which was found to increase significantly as the network density increases. The results presented here indicate that C-V measurement is a powerful tool to assess the electrical performance and to investigate the carrier transport mechanism of TFTs based on carbon nanotubes.

**Electronic supplementary material:**

The online version of this article (doi:10.1186/s11671-015-0999-8) contains supplementary material, which is available to authorized users.

## Background

Owing to its extremely large carrier mean free path and high mobility, semiconducting single-wall carbon nanotube (sSWCNT) is considered as one of the promising candidates for beyond-silicon electronics [1–3]. However, despite the tremendous progress made in individual sSWCNT electronic device and circuit research, scalable fabrication and integration of large quantities of devices with uniform performance remain to be greatly challenging, largely due to the structure heterogeneity of carbon nanotubes and difficulties in assembling them with nanoscale precision [4]. On the other hand, using random networks of carbon nanotubes for applications in thin-film transistors (TFTs) has recently shown great promise. Such devices offer mechanical flexibility and optical transparency and can be easily fabricated in a scalable fashion with far superior device performance and long-term air-stability compared with amorphous silicon or organic semiconductors [5–11]. Among various approaches used for assembling random carbon nanotube networks, solution-processed semiconductor-enriched carbon nanotubes possess additional advantages of low-cost and room temperature processes [6, 8–10, 12]. With the ongoing pavement towards reliable dispersion of longer nanotubes with higher purity separation process [13], large-scale, high-performance, flexible carbon nanotube TFTs have already found wide applications in compliant integrated circuits, organic light-emitting displays, and electronic skins [5, 9, 11, 14].

One important device parameter used for assessing the electrical performance of TFT is its field-effect mobility. Precise evaluation of the field-effect mobility is crucial for the fair comparison of device performance between different material platforms. In order to extract the mobility, information about the gate capacitance is needed, which reflects the electrostatic coupling between the channel semiconductor and the planar gate electrode. For carbon nanotube transistors, the gate capacitances are typically calculated from either an ideal parallel-plate model or a more rigorous cylindrical model by considering the electrostatic coupling between nanotubes [5, 7, 15, 16]. The former apparently overestimates the gate capacitance due to low coverage of nanotubes in the channel and thereby underestimates the device mobility, while the latter sometimes overestimates the mobility due to uncertainty in determining tube diameter and density for a random network. In principle, the gate capacitance can be experimentally determined from the capacitance-voltage (C-V) characteristics of the transistors, which should provide a more precise evaluation of the device performance. Additionally, C-V measurement is a powerful tool to get deep physical insights of the electronic performance of metal-insulator-semiconductor (MIS) structures [17, 18].

Here, in this paper, we report the C-V measurements on solution-processed carbon nanotube TFTs with various network densities. Based on the C-V characteristics, field-effect mobility of the devices was accurately assessed and compared with values estimated using both parallel-plate and cylindrical capacitance models. In addition, the C-V characteristics were measured at different frequencies to allow further extraction of the interface trap density, which could shed light on the quality and cleanness of the interface between the solution-processed carbon nanotubes and the gate dielectric layer.

## Methods

**Fig. 1:**
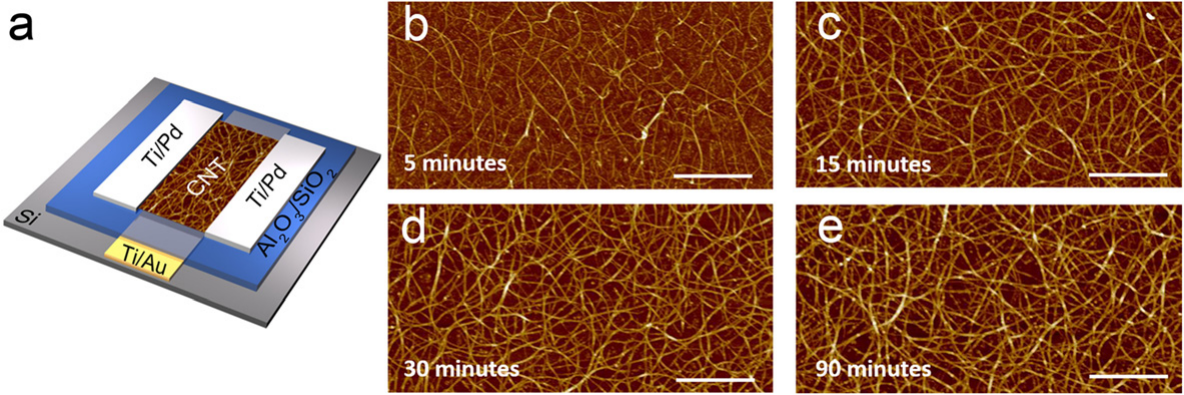
Thin-film transistors based on solution-processed random networks of semiconducting carbon nanotubes. **a** Schematic of the device structure. **b**–**e** AFM images of the networks deposited by immersing the substrate into 0.01 mg ml^−1^ sSWCNT solution for 5, 15, 30, and 90 min, respectively. *Scale bars* in **b**–**e**, 1 μm

High-purity semiconducting SWCNTs with a purity of approximated 99 % (purchased from NanoIntegris, Inc.) were used as the channel semiconductor. The device structure of the TFTs used in this study is presented in Fig. 1a, which incorporates a local back-gate. Both overlap gate and underlap gate devices were fabricated on the same substrate. The detailed fabrication process resembles the one reported in our previous publications [4, 8]. Briefly, electron beam (e-beam) evaporated Ti/Au (5/50 nm) was patterned as the gate (G) electrode using photolithography, followed by deposition of 25 nm of Al_2_O_3_ and 10 nm of SiO_2_ as the gate dielectric layer using atomic layer deposition and e-beam evaporation, respectively. Uniform networks of sSWCNTs were obtained by functionalizing the substrate surface with amine-group (immersing in 0.1 g ml^−1^ poly-l-lysine solution for 5 min) before immersing into 0.01 mg ml^−1^ sSWCNT solutions. The density of the carbon nanotube network can be effectively controlled by adjusting the deposition time as shown in Fig. 1b–e. The atomic force microscopy (AFM) images indicate a monotonic increase in network density as the deposition time increases from 5 to 90 min. On top of the carbon nanotube network, source/drain (S/D) electrodes, composed of 0.5 nm Ti and 50 nm Pd, were patterned and the carbon nanotubes outside the channel region were subsequently etched away using O_2_ plasma. Finally, via holes were patterned and opened by HF etching to expose the gate probing pads.

Both current-voltage (I-V) and C-V characteristics were measured using an Agilent B1500A Semiconductor Device Parameter Analyzer. For I-V measurements, devices with overlap gate configuration (G completely overlaps with S/D) were used in order to eliminate the un-gated region and minimize the access resistance. For C-V measurements, underlap gate structure was adopted (gate length *L*_g_ of 3, 8, and 16 μm for channel length *L* of 4, 10, and 20 μm, respectively) to minimize the parasitic capacitance.

## Results and Discussion

### I-V Characteristics

**Fig. 2:**
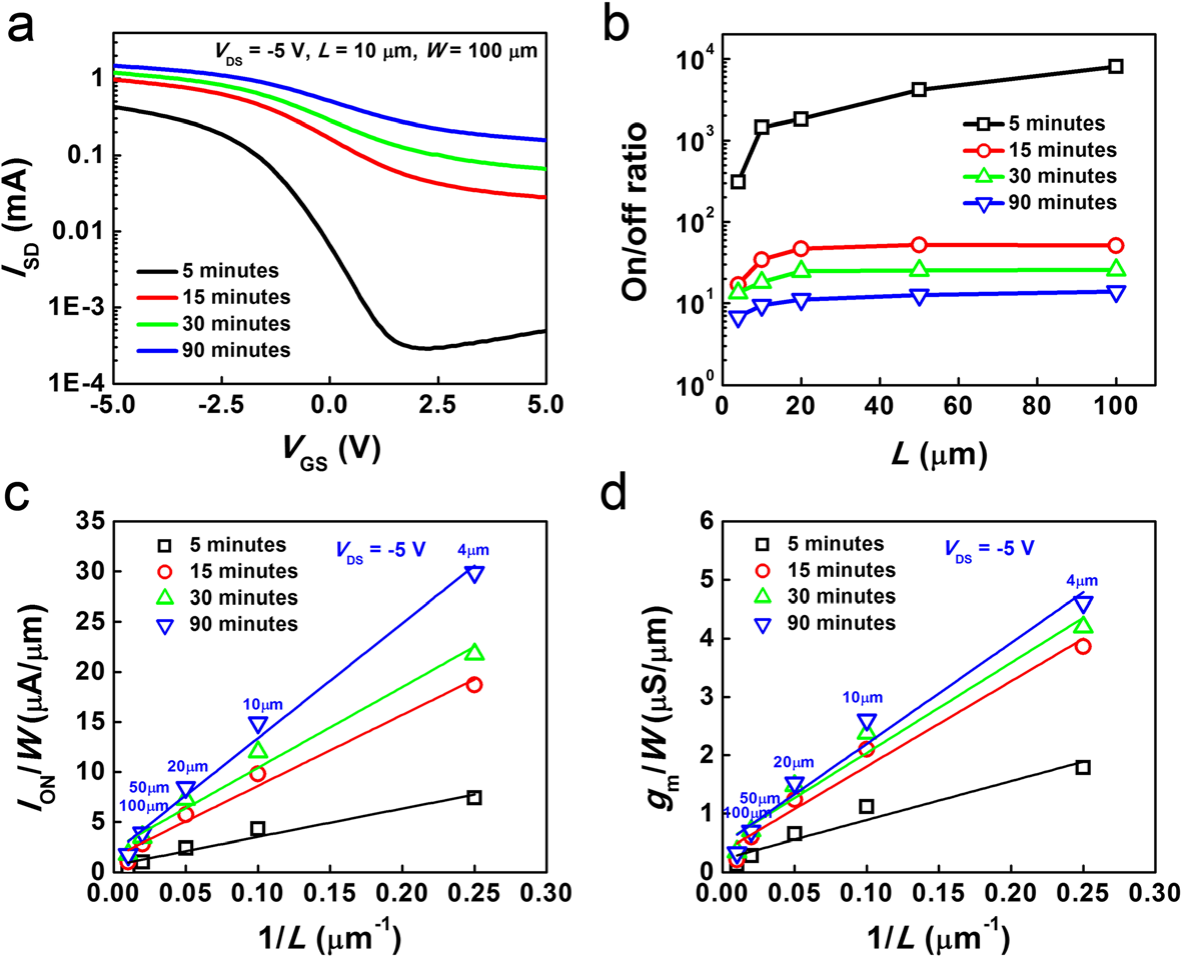
*I*_SD_-*V*_GS_ characteristics of the TFTs with overlap gates. **a** *I*_SD_-*V*_GS_ curves measured at a VDS of −5 V for devices (*L* = 10 μm, *W* = 100 μm) with different deposition times and thereby different network densities. **b**–**d** On/off ratio vs. *L* (**b**), width normalized on-current vs. 1/*L* (**c**), and width normalized transconductance vs. 1/*L* (**d**) for devices with different network densities

I-V characterization was performed on the above-described solution-processed carbon nanotube TFTs with various channel lengths and network densities (as the nanotube deposition time increases from 5 up to 90 min). Figure 2a shows the representative transfer characteristics (*I*_SD_-*V*_GS_) of devices with different nanotube network densities measured at *V*_DS_ = −5 V. All devices presented in Fig. 2a have the same channel length (*L*) and width (*W*) of 10 and 100 μm, respectively. All *I*_SD_-*V*_GS_ curves show p-type behavior with gradually decreasing on/off current ratio (*I*_on_/*I*_off_) and increasing on-current (*I*_on_) as the nanotube density increases. More systematic measurements were carried out on devices with different channel lengths and the results are summarized in Fig. 2b–d. Device performance metrics of the TFTs, including *I*_on_/*I*_off_, unit width normalized on-current (*I*_on_/*W*) and transconductance (*g*_m_/*W*), are extracted from the measured *I*_SD_-*V*_GS_ curves (shown in Additional file 1: Figure S1 of the Electronic Supplementary Material) to allow the analysis of their dependence on channel length and network density.

For all the device metrics listed above, the most significant change occurs as the deposition time increases from 5 to 15 min, in accordance to the evolution of network density. According to Fig. 2b, devices with lower network density and larger channel length tend to have higher on/off ratio (up to 10^4^), which is resulted from the lower probability for metallic nanotubes to form percolating pathways between the S/D in looser network and long-channel devices. On the other hand, lower network density also leads to significant decrease in *I*_on_ and *g*_m_ as shown in Fig. 2c, d. This trade-off is an important design consideration for optimizing device performance of carbon nanotube TFTs targeting different applications. According to our previous studies, low network density long-channel devices, with high *I*_on_/*I*_off_, can be used for compliant digital electronics or as switches in backplane, while high-density short-channel ones are ideal for high-frequency applications [8, 9, 14]. Additionally, both *I*_on_/*W* and *g*_m_/*W* are approximately proportional to the reciprocal of channel length (1/*L*), which is in agreement with conventional field-effect transistor operation theory and also indicates the excellent uniformity of nanotube networks in our TFTs. The TFTs with shortest channels exhibit on-current and transconductance as high as ~30 μA μm^−1^ and ~4.5 μS μm^−1^, respectively, which is respectable performance for a solution-processed approach.

### C-V Characteristics

**Fig. 3:**
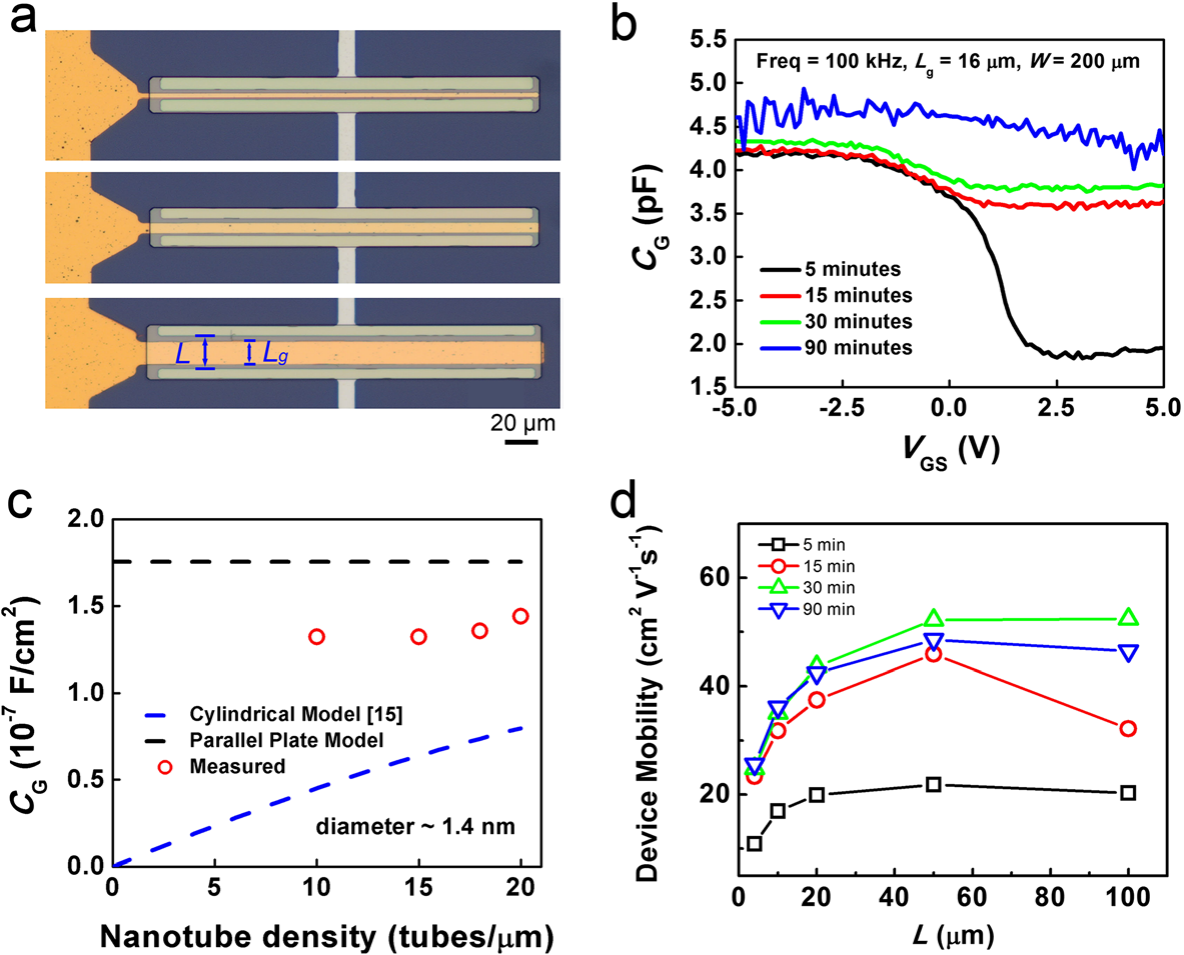
C-V characteristics measured from nanotube TFTs with underlap gates. **a** Optical micrographs of underlap gate devices with different gate length of 3 μm (*top*), 8 μm (*middle*), and 16 μm (*bottom*). **b** C-V characteristics for devices (*L* = 20 μm, *W* = 200 μm) with different network densities measured at a frequency of 100 kHz. **c** Unit-area gate capacitance extracted from the measured C-V characteristics (*red circle*), calculated from parallel-plate model (*black dashed line*), and calculated from cylindrical model (*blue dash line*) plotted as a function of nanotube network density. **d** Device mobility extracted using the experimentally measured gate capacitance, plotted as a function of channel length and for different deposition times

In order to minimize the effect of parasitic capacitance on C-V measurements, devices with underlap gate electrode were used whose optical microscope images are shown in Fig. 3a. Such devices have channel lengths *L* (i.e., the separation between S/D electrodes) of 4, 10, and 20 μm, while the underlapped gate fingers have lengths *L*_g_ of 3, 8, and 16 μm, respectively. *L*_g_ instead of *L* was used when calculating the area for unit-area gate capacitance.

C-V characteristics of devices with different channel lengths and nanotube network densities were measured over a frequency range of 2 kHz~1 MHz and the results are shown in Additional file 1: Figures S2 and S3. Figure 3b shows the representative C-V curves of TFTs (*L*_g_ = 16 μm, *W* = 200 μm) with different network densities measured at a frequency of 100 kHz. The measured C-V curves in Fig. 3b generally resemble the trend observed in the I-V curves (Fig. 2), which is understandable considering the fact that gate modulation decreases with increasing deposition time and nanotube density as a result of more metallic pathways.

Additional file 1: Figure S4b shows the on-state capacitance (*V*_GS_ = −5 V) plotted as a function of channel area (*L*_g_ × *W*), where the unit-area gate capacitance can be deduced from the slope of linear fit of the data points. The experimentally determined gate capacitance from Additional file 1: Figure S4b was summarized and plotted as a function of nanotube density in Fig. 3c. Also presented are the parallel-plate capacitance (black dashed line), which was also experimentally measured using on-chip capacitors fabricated with the same dielectric layer as the TFTs (see Additional file 1: Figure S4a), and that calculated from the cylindrical model (blue dashed lines) using the equation reported in the literature [15] with an estimated average nanotube diameter of 1.4 nm. From the results, it is obvious that the parallel-plate model overestimates the gate capacitance while the cylindrical model underestimates it. This manifests the necessity of using C-V characteristics to accurately evaluate the gate capacitance and mobility.

### Analysis of C-V Characteristics

Based on the measured gate capacitance, the field-effect mobility of the devices can be extracted using the following equation:

$$ \mu =\frac{L}{V_d{C}_{\mathrm{ox}}W}\frac{d{I}_g}{d{V}_g}=\frac{L}{V_d{C}_{\mathrm{ox}}}\frac{g_m}{W}. $$

Figure 3d shows the mobility as a function of channel length for devices with different deposition times. For all measured devices, the mobility increases initially with channel length and then saturates at a channel length of around 50 μm. For short-channel devices, the current is mostly limited by nanotube-electrode contact resistance instead of channel resistance. As a result, increase in channel length would lead to increase in the extracted field-effect mobility, until *L* ~ 50 μm when the channel resistance begins to take the dominance over contact resistance. The highest mobility of our devices is ~50 cm^2^ V^−1^ s^−1^, orders of magnitude higher than that of amorphous silicon and most organic semiconductors, making sSWCNTs ideal candidate for high-performance, solution-processed flexible TFTs.

**Fig. 4:**
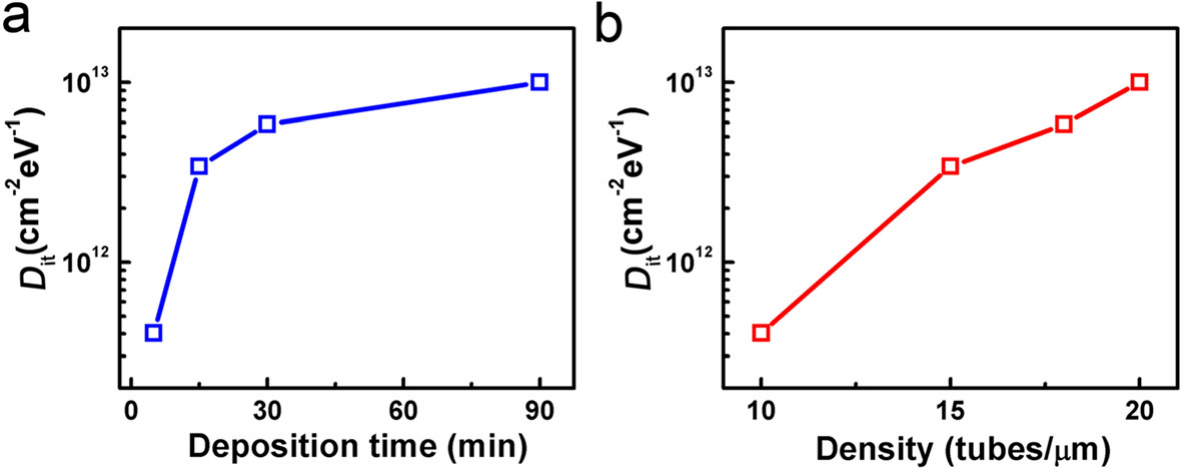
Interface trap density of solution-processed carbon nanotube TFTs. **a**, **b** Relationship between extracted interface trap density and deposition time (**a**) and nanotube network density (**b**)

Further analysis of the C-V data can lead to deeper physical insights of the device performance, such as the interface trap density (*D*_it_) at the nanotube and gate dielectric interface. The interface trap density can be extracted from C-V characteristics measured at high and low frequencies using the equation below [8, 17]:

$$ {D}_{\mathrm{it}}=\frac{C_{\mathrm{LF}}-{C}_{\mathrm{HF}}}{q\left(1-\frac{C_{\mathrm{LF}}}{C_{\mathrm{ox}}}\right)\left(1-\frac{C_{\mathrm{HF}}}{C_{\mathrm{ox}}}\right)LW} $$

where *C*_LF_ and *C*_HF_ are the low- and high-frequency capacitance, respectively, and *q* = 1.6 × 10^−19^*C*. Figure 4a, b shows the extracted *D*_it_ plotted as a function of deposition time and nanotube network density, respectively. The results indicate that the interface trap density could increase drastically as the deposition time/network density increases. Because the nanotube networks in our devices are assembled using a solution-based process, longer deposition time may lead to more residues from the solvent such as excessive surfactant or dust particles, which could degrade the cleanness of surface of the resulting carbon nanotube network, leading to significantly more interface traps. Because *D*_it_ has negative impact on the overall device performance (e.g., lead to a worse subthreshold slope), it is important to keep it as low as possible. It should be pointed out that the real *D*_it_ is underestimated using the strategy here due to the low coverage of sSWCNTs in the channel region. Nevertheless, our results show that C-V characterization can be a powerful tool to unveil more insights of the carbon nanotube TFT performance.

## Conclusions

In summary, we have fabricated high-performance TFTs using solution-processed sSWCNT network as the channel material. Systematic I-V and C-V characterizations were performed to study the relationship between various device performance metrics and nanotube density. We have also shown that the C-V measurements could lead to more accurate assessment of gate capacitance which in turn results in the evaluation of device mobility with a higher accuracy than other most widely adopted models. Finally, interface trap densities were also extracted from the C-V measurements and the results indicate that longer nanotube deposition time would lead to significantly more interface traps. The results presented here indicate that C-V measurement is a powerful means for the accurate evaluation of the performance of nanotube TFTs and the investigation of their carrier transport mechanism, both of which are important for further device optimization.

## Additional file

Additional file 1: Figure S1.
*I*SD-*V*GS curves for TFTs with different channel lengths and deposition time of (a) 5 min; (b) 15 min; (c) 30 min, and (d) 90 min. **Figure S2.** Capacitance-voltage characteristics for devices with different channel lengths and deposition time of (a) 5 min, (b) 15 min, (c) 30 min, and (d) 90 min, measured at a frequency of 100 kHz. **Figure S3.** Capacitance-voltage (C-V) curves at different frequencies (2 kHz –1 MHz) of the TFT with a deposition time of 5 min. **Figure S4.** Measured capacitance *vs.* effective area for parallel capacitors (a) and carbon nanotube TFTs with underlapped gate electrodes (b). The insets show the optical micrograph of a parallel capacitor and an underlap gate TFT, where the effective capacitance areas are indicated.

## References

[1] Franklin AD, Luisier M, Han S-J, Tulevski G, Breslin CM, Gignac L (2012). Sub-10 nm carbon nanotube transistor. Nano Lett.

[2] Javey A, Guo J, Wang Q, Lundstrom M, Dai H (2003). Ballistic carbon nanotube field-effect transistors. Nature.

[3] Franklin AD, Chen Z (2010). Length scaling of carbon nanotube transistors. Nat Nanotechnol.

[4] Wang C, Takei K, Takahashi T, Javey A (2013). Carbon nanotube electronics—moving forward. Chem Soc Rev.

[5] Cao Q, Kim H-S, Pimparkar N, Kulkarni JP, Wang C, Shim M (2008). Medium-scale carbon nanotube thin-film integrated circuits on flexible plastic substrates. Nature.

[6] Engel M, Small JP, Steiner M, Freitag M, Green AA, Hersam MC (2008). Thin film nanotube transistors based on self-assembled, aligned, semiconducting carbon nanotube arrays. Acs Nano..

[7] Snow E, Novak J, Campbell P, Park D (2003). Random networks of carbon nanotubes as an electronic material. Appl Phys Lett.

[8] Wang C, Chien J-C, Takei K, Takahashi T, Nah J, Niknejad AM (2012). Extremely bendable, high-performance integrated circuits using semiconducting carbon nanotube networks for digital, analog, and radio-frequency applications. Nano Lett.

[9] Wang C, Zhang J, Ryu K, Badmaev A, De Arco LG, Zhou C (2009). Wafer-scale fabrication of separated carbon nanotube thin-film transistors for display applications. Nano Lett.

[10] Wang C, Zhang J, Zhou C (2010). Macroelectronic integrated circuits using high-performance separated carbon nanotube thin-film transistors. Acs Nano.

[11] Sun D, Timmermans MY, Tian Y, Nasibulin AG, Kauppinen EI, Kishimoto S (2011). Flexible high-performance carbon nanotube integrated circuits. Nat Nanotechnol.

[12] Arnold MS, Green AA, Hulvat JF, Stupp SI, Hersam MC (2006). Sorting carbon nanotubes by electronic structure using density differentiation. Nat Nanotechnol.

[13] Jariwala D, Sangwan VK, Lauhon LJ, Marks TJ, Hersam MC (2013). Carbon nanomaterials for electronics, optoelectronics, photovoltaics, and sensing. Chem Soc Rev.

[14] Wang C, Hwang D, Yu Z, Takei K, Park J, Chen T (2013). User-interactive electronic skin for instantaneous pressure visualization. Nat Mater.

[15] Cao Q, Xia M, Kocabas C, Shim M, Rogers JA, Rotkin SV (2007). Gate capacitance coupling of singled-walled carbon nanotube thin-film transistors. Appl Phys Lett.

[16] Kang SJ, Kocabas C, Ozel T, Shim M, Pimparkar N, Alam MA (2007). High-performance electronics using dense, perfectly aligned arrays of single-walled carbon nanotubes. Nat Nanotechnol.

[17] Streetman BG (2006). Solid state electronic devices.

[18] Takei K, Kapadia R, Fang H, Plis E, Krishna S, Javey A. High quality interfaces of InAs-on-insulator field-effect transistors with ZrO2 gate dielectrics. Appl Phys Lett. 2013;102(15). doi:10.1063/1.48027792.

