# Supplementary material for: Capacitance-Voltage Characteristics of Thin-film Transistors Fabricated with Solution-Processed Semiconducting Carbon Nanotube Networks
Source: Nanoscale Res Lett. 2015 Jul 15;10:291. doi: 10.1186/s11671-015-0999-8 (PMC4501368; doi:10.1186/s11671-015-0999-8)
Supplement: Additional file 1: Figure S1. — ISD-VGS curves for TFTs with different channel lengths and deposition time of (a) 5 min; (b) 15 min; (c) 30 min, and (d) 90 min. Figure S2. Capacitance-voltage characteristics for devices with different channel lengths and deposition time of (a) 5 min, (b) 15 min, (c) 30 min, and (d) 90 min, measured at a frequency of 100 kHz. Figure S3. Capacitance-voltage (C-V) curves at different frequencies (2 kHz –1 MHz) of the TFT with a deposition time of 5 min. Figure S4. Measured capacitance vs. effective area for parallel capacitors (a) and carbon nanotube TFTs with underlapped gate electrodes (b). The insets show the optical micrograph of a parallel capacitor and an underlap gate TFT, where the effective capacitance areas are indicated. [file 11671_2015_999_MOESM1_ESM.pdf]

Additional Information

Capacitance-voltage characteristics of thin-film transistors fabricated with solution-processed semiconducting carbon nanotube networks

Le Cai[1], Suoming Zhang[1], Jinshui Miao[1], Qinqin Wei[1], Chuan Wang[1]*

[1] *Department of Electrical and Computer Engineering, Michigan State University, East Lansing, Michigan 48824, USA*

E-mail: cwang@msu.edu

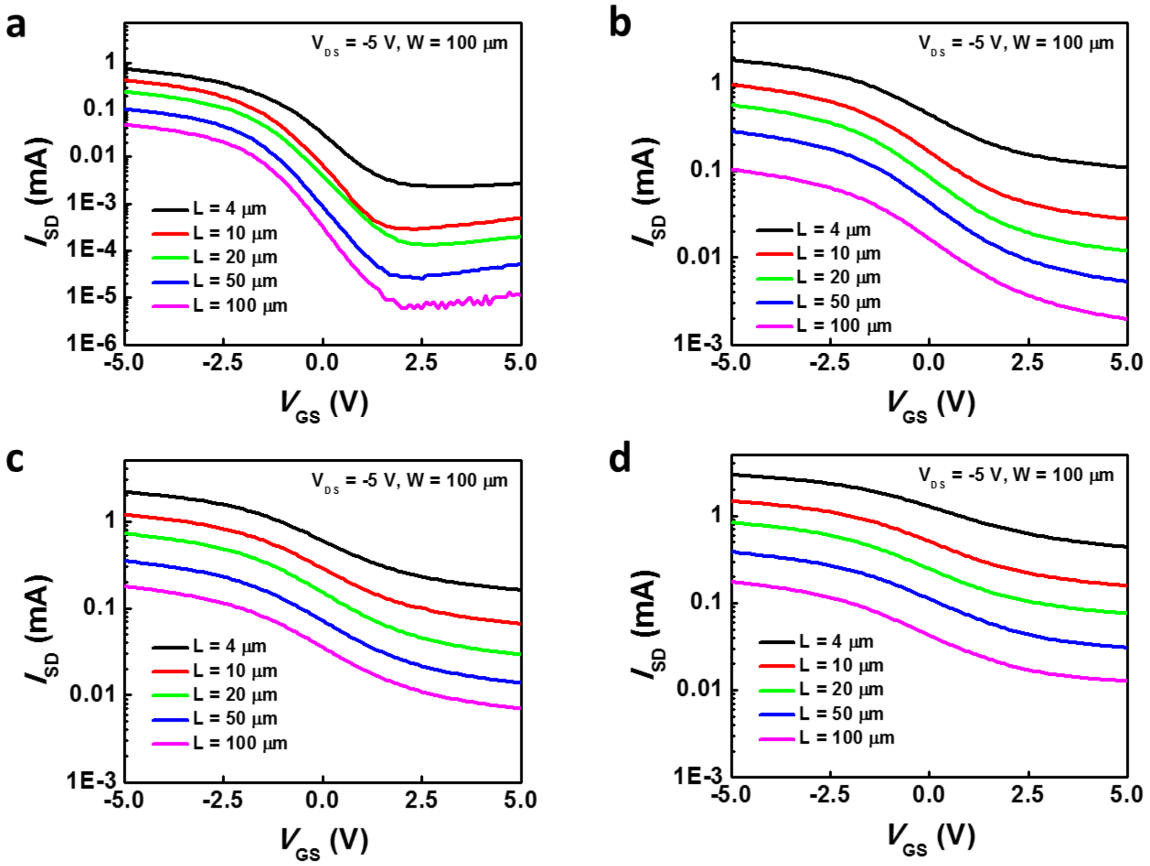

Figure S1. I_{SD}-V_{GS} curves for TFTs with different channel lengths and deposition time of (a) 5 min; (b) 15 min; (c) 30 min, and (d) 90 min.

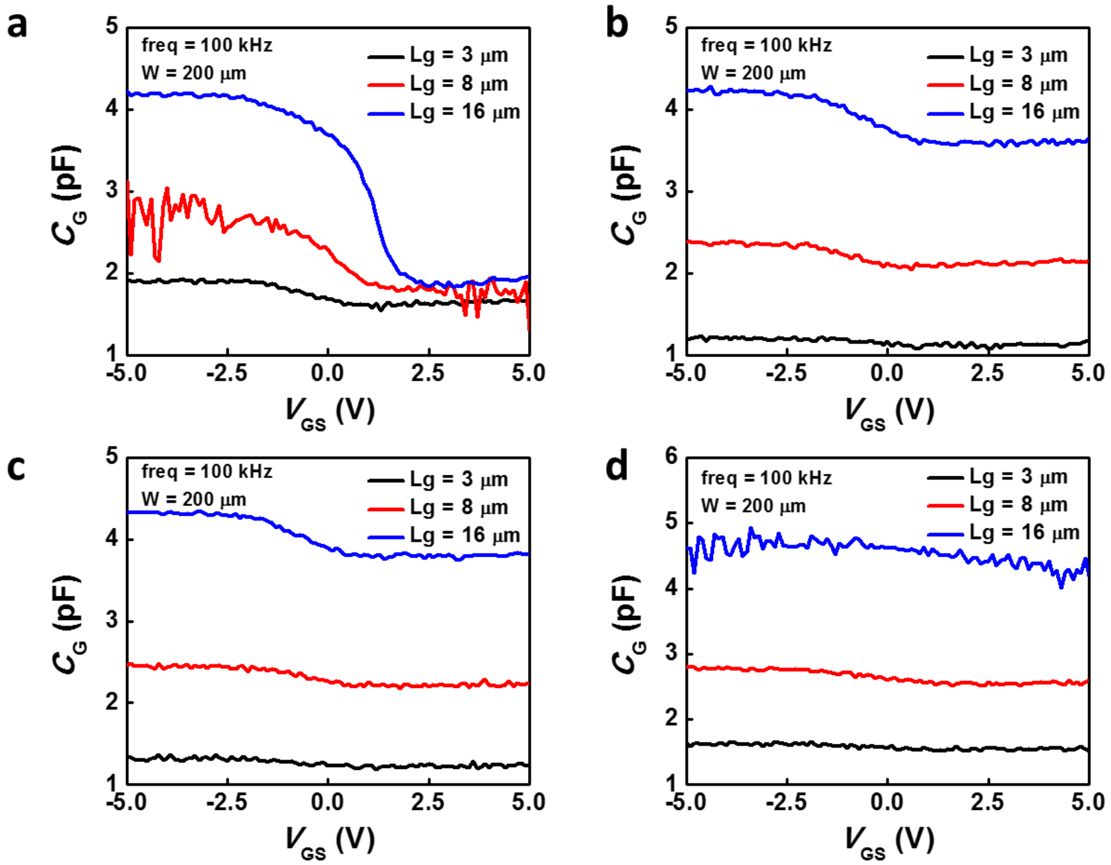

Figure S2. Capacitance-voltage characteristics for devices with different channel lengths and deposition time of (a) 5 min, (b) 15 min, (c) 30 min, and (d) 90 min, measured at a frequency of 100 kHz.

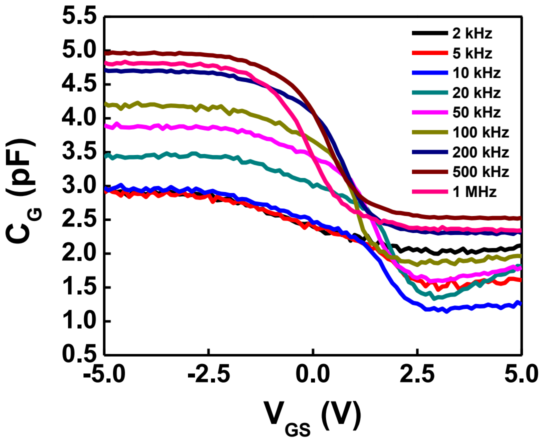

Figure S3. Capacitance-voltage (C-V) curves at different frequencies (2 kHz – 1 MHz) of the TFT with a deposition time of 5 min.

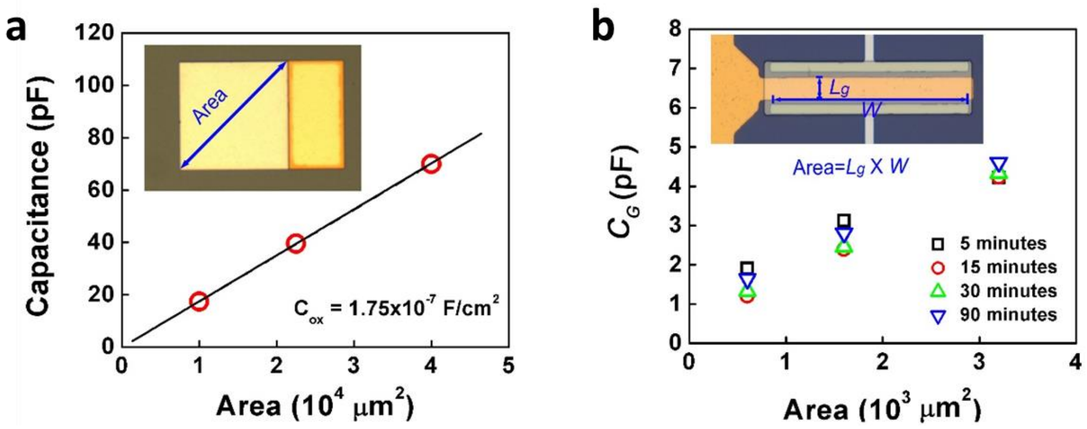

Figure S4. Measured capacitance *vs.* effective area for parallel capacitors (a) and carbon nanotube TFTs with underlapped gate electrodes (b). The insets show the optical micrograph of a parallel capacitor and an underlap gate TFT, where the effective capacitance areas are indicated.
